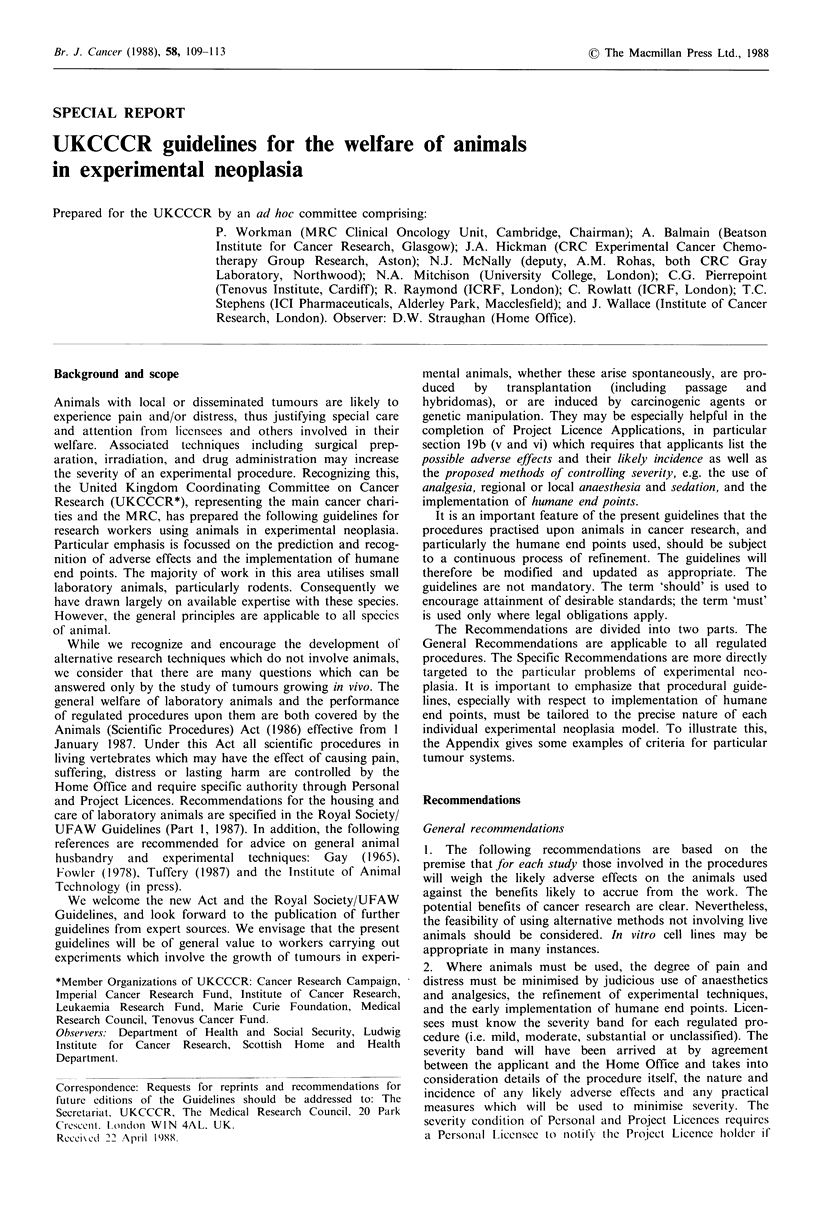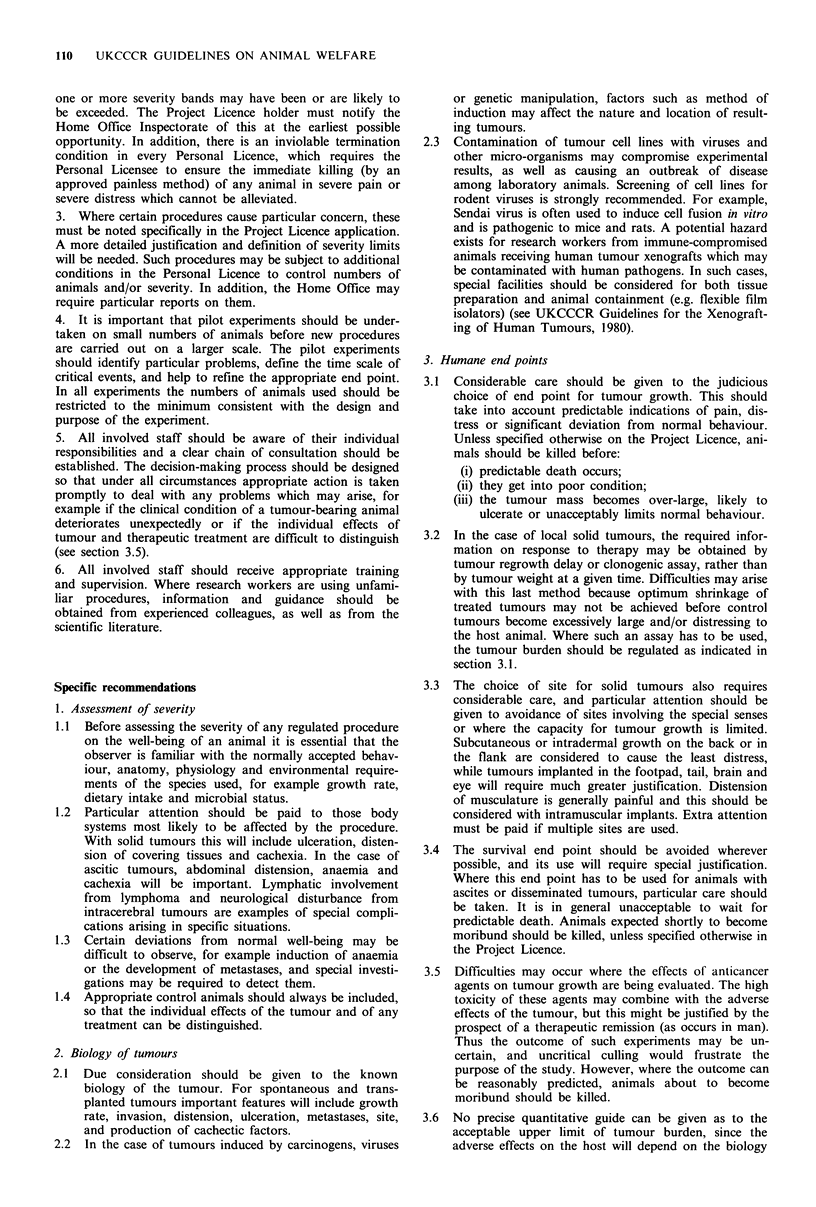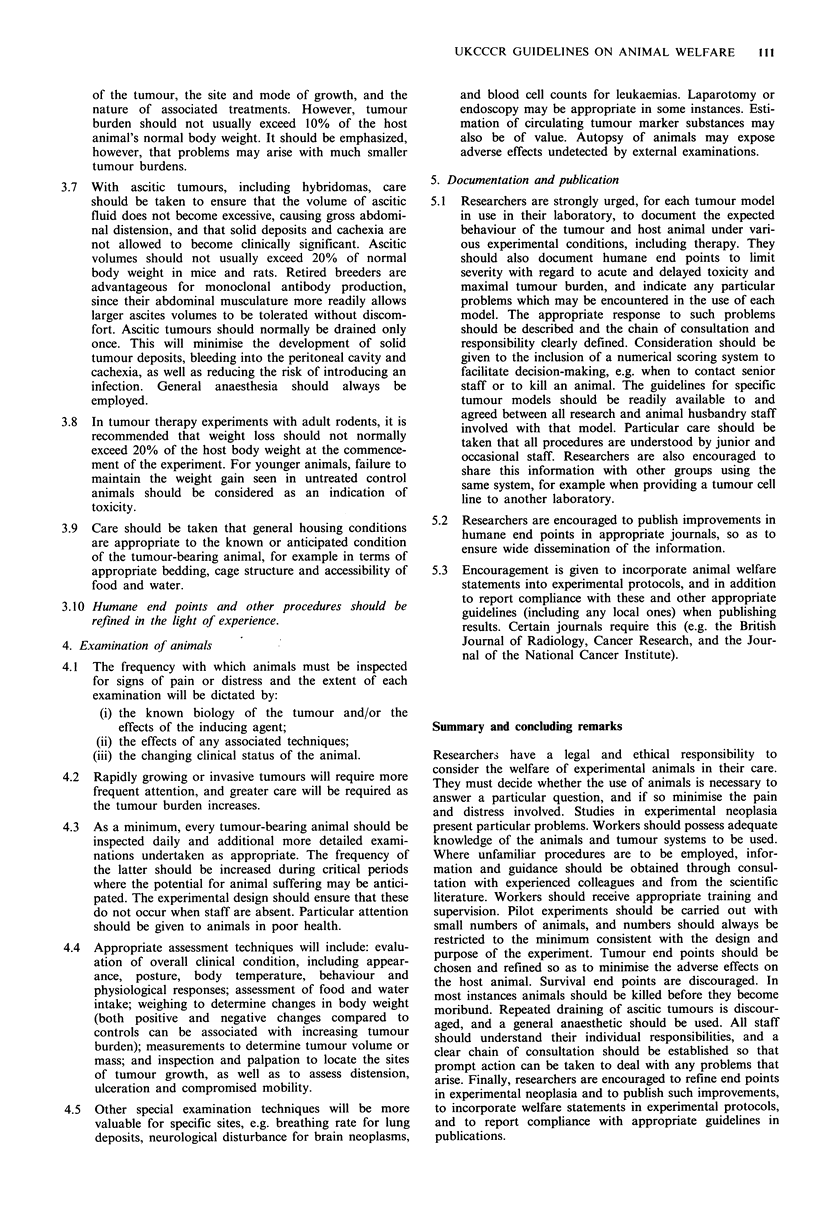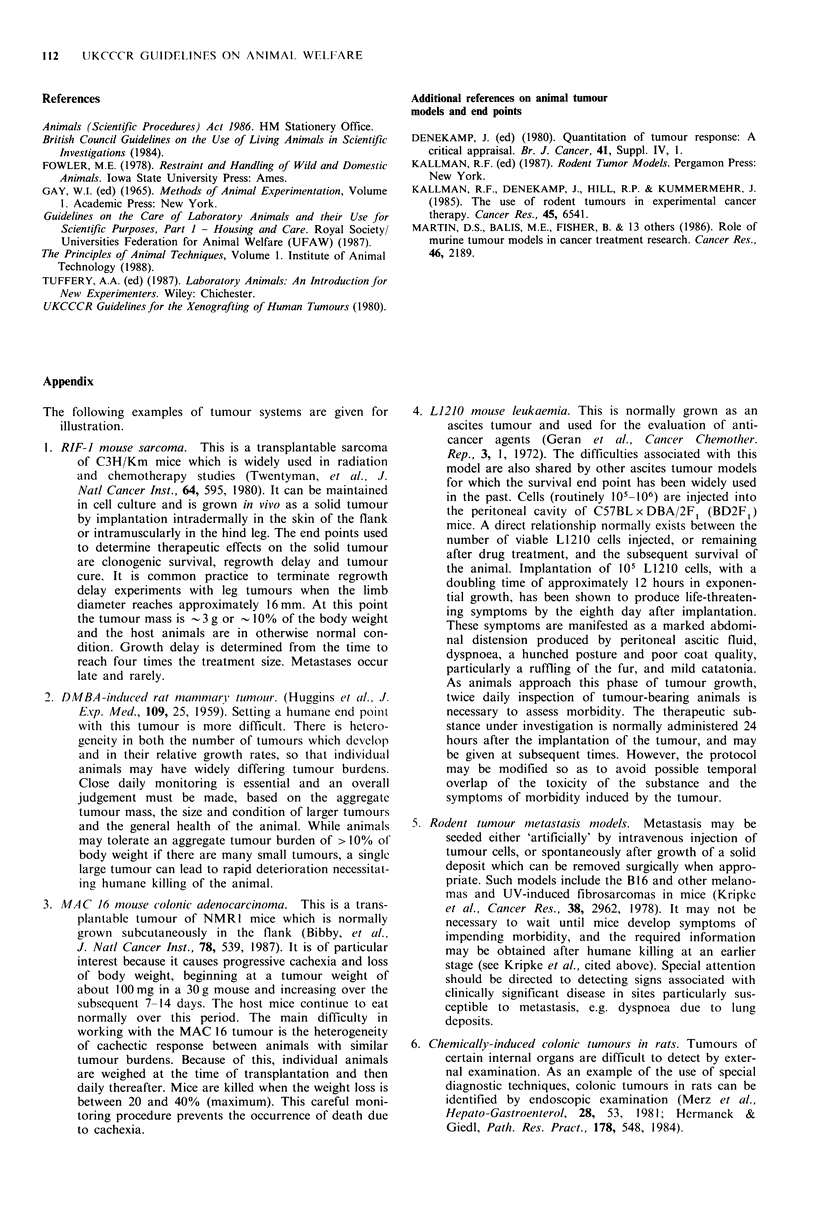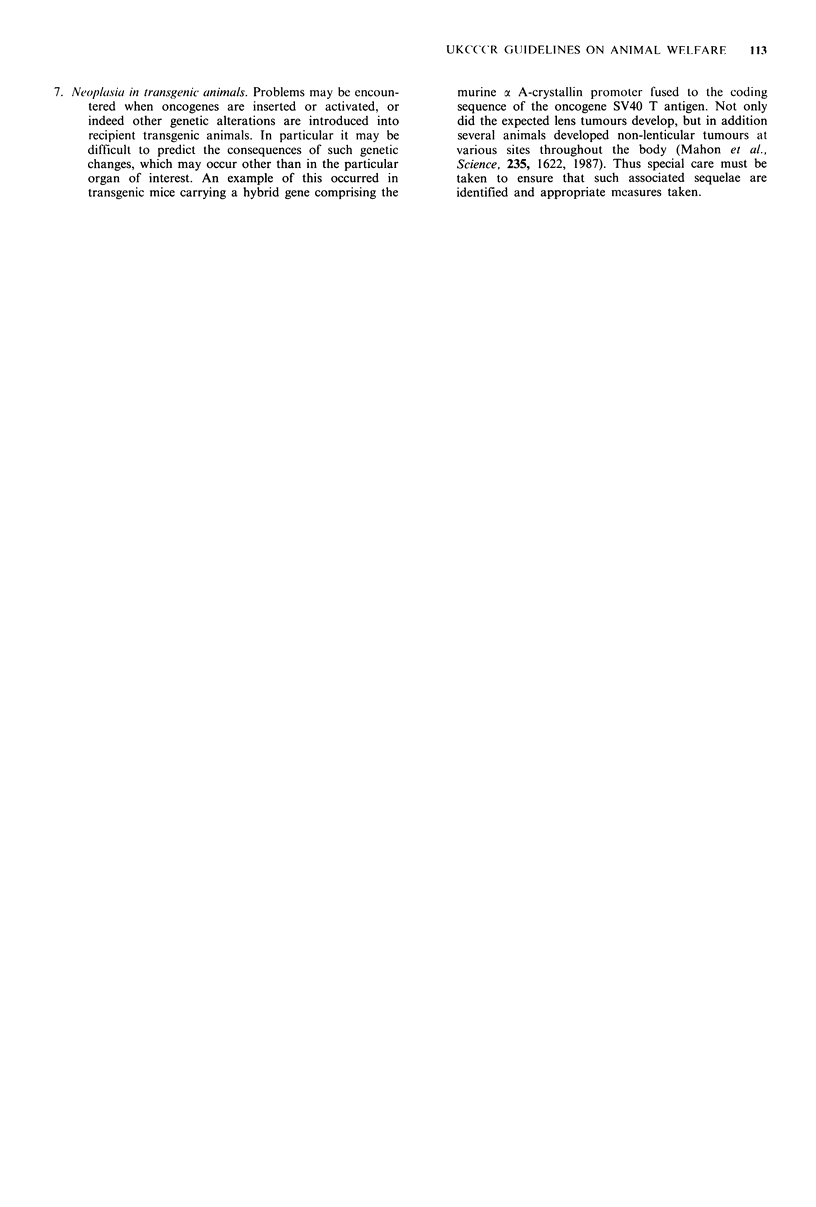# UKCCCR guidelines for the welfare of animals in experimental neoplasia.

**DOI:** 10.1038/bjc.1988.174

**Published:** 1988-07

**Authors:** 


 © The Macmillan Press Ltd., 1988

SPECIAL REPORT

### UKCCCR guidelines for the welfare of animals in experimental neoplasia

Prepared for the UKCCCR by an *ad hoc* committee comprising:

P. Workman (MRC Clinical Oncology Unit, Cambridge, Chairman); A. Balmain (Beatson Institute for Cancer Research, Glasgow); J.A. Hickman (CRC Experimental Cancer Chemotherapy Group Research, Aston); N.J. McNally (deputy, A.M. Rohas, both CRC Gray Laboratory, Northwood); N.A. Mitchison (University College, London); C.G. Pierrepoint (Tenovus Institute, Cardiff); R. Raymond (ICRF, London); C. Rowlatt (ICRF, London); T.C. Stephens (ICI Pharmaceuticals, Alderley Park, Macclesfield); and J. Wallace (Institute of Cancer Research, London). Observer: D.W. Straughan (Home Office).

### Background and scope

Animals with local or disseminated tumours are likely to experience pain and/or distress, thus justifying special care and attention from licensees and others involved in their welfare. Associated techniques including surgical preparation, irradiation, and drug administration may increase the severity of an experimental procedure. Recognizing this, the United Kingdom Coordinating Committee on Cancer Research (UKCCCR*), representing the main cancer charities and the MRC, has prepared the following guidelines for research workers using animals in experimental neoplasia. Particular emphasis is focussed on the prediction and recognition of adverse effects and the implementation of humane end points. The majority of work in this area utilises small laboratory animals, particularly rodents. Consequently we have drawn largely on available expertise with these species. However, the general principles are applicable to all species of animal.

While we recognize and encourage the development of alternative research techniques which do not involve animals, we consider that there are many questions which can be answered only by the study of tumours growing *in vivo*. The general welfare of laboratory animals and the performance of regulated procedures upon them are both covered by the Animals (Scientific Procedures) Act (1986) effective from 1 January 1987. Under this Act all scientific procedures in living vertebrates which may have the effect of causing pain, suffering, distress or lasting harm are controlled by the Home Office and require specific authority through Personal and Project Licences. Recommendations for the housing and care of laboratory animals are specified in the Royal Society/UFAW Guidelines (Part 1, 1987). In addition, the following references are recommended for advice on general animal husbandry and experimental techniques: Gay (1965), Fowler (1978), Tuffery (1987) and the Institute of Animal Technology (in press).

We welcome the new Act and the Royal Society/UFAW Guidelines, and look forward to the publication of further guidelines from expert sources. We envisage that the present guidelines will be of general value to workers carrying out experiments which involve the growth of tumours in experimental animals, whether these arise spontaneously, are produced by transplantation (including passage and hybridomas), or are induced by carcinogenic agents or genetic manipulation. They may be especially helpful in the completion of Project Licence Applications, in particular section 19b (v and vi) which requires that applicants list the *possible adverse effects* and their *likely incidence* as well as the *proposed methods of controlling severity,* e.g. the use of *analgesia,* regional or local *anaesthesia* and *sedation,* and the implementation of *humane end points.*

It is an important feature of the present guidelines that the procedures practised upon animals in cancer research, and particularly the humane end points used, should be subject to a continuous process of refinement. The guidelines will therefore be modified and updated as appropriate. The guidelines are not mandatory. The term 'should' is used to encourage attainment of desirable standards; the term 'must' is used only where legal obligations apply.

The Recommendations are divided into two parts. The General Recommendations are applicable to all regulated procedures. The Specific Recommendations are more directly targeted to the particular problems of experimental neoplasia. It is important to emphasize that procedural guidelines, especially with respect to implementation of humane end points, must be tailored to the precise nature of each individual experimental neoplasia model. To illustrate this, the Appendix gives some examples of criteria for particular tumour systems.

### Recommendations

### *General recommendations*

1. The following recommendations are based on the premise that *for each study* those involved in the procedures will weigh the likely adverse effects on the animals used against the benefits likely to accrue from the work. The potential benefits of cancer research are clear. Nevertheless, the feasibility of using alternative methods not involving live animals should be considered. *In vitro* cell lines may be appropriate in many instances.

2. Where animals must be used, the degree of pain and distress must be minimised by judicious use of anaesthetics and analgesics, the refinement of experimental techniques, and the early implementation of humane end points. Licensees must know the severity band for each regulated procedure (i.e. mild, moderate, substantial or unclassified). The severity band will have been arrived at by agreement between the applicant and the Home Office and takes into consideration details of the procedure itself, the nature and incidence of any likely adverse effects and any practical measures which will be used to minimise severity. The severity condition of Personal and Project Licences requires a Personal Licensee to notify the Project Licence holder if

*Member Organizations of UKCCCR: Cancer Research Campaign, Imperial Cancer Research Fund, Institute of Cancer Research, Leukaemia Research Fund, Marie Curie Foundation, Medical Research Council, Tenovus Cancer Fund.
*Observers:* Department of Health and Social Security, Ludwig Institute for Cancer Research, Scottish Home and Health Department.

Correspondence: Requests for reprints and recommendations for future editions of the Guidelines should be addressed to: The Secretariat, UKCCCR, The Medical Research Council, 20 Park Crescent, London W1N 4AL, UK.
Received 22 April 1988.

one or more severity bands may have been or are likely to be exceeded. The Project Licence holder must notify the Home Office Inspectorate of this at the earliest possible opportunity. In addition, there is an inviolable termination condition in every Personal Licence, which requires the Personal Licensee to ensure the immediate killing (by an approved painless method) of any animal in severe pain or severe distress which cannot be alleviated.

3. Where certain procedures cause particular concern, these must be noted specifically in the Project Licence application. A more detailed justification and definition of severity limits will be needed. Such procedures may be subject to additional conditions in the Personal Licence to control numbers of animals and/or severity. In addition, the Home Office may require particular reports on them.

4. It is important that pilot experiments should be undertaken on small numbers of animals before new procedures are carried out on a larger scale. The pilot experiments should identify particular problems, define the time scale of critical events, and help to refine the appropriate end point. In all experiments the numbers of animals used should be restricted to the minimum consistent with the design and purpose of the experiment.

5. All involved staff should be aware of their individual responsibilities and a clear chain of consultation should be established. The decision-making process should be designed so that under all circumstances appropriate action is taken promptly to deal with any problems which may arise, for example if the clinical condition of a tumour-bearing animal deteriorates unexpectedly or if the individual effects of tumour and therapeutic treatment are difficult to distinguish (see section 3.5).

6. All involved staff should receive appropriate training and supervision. Where research workers are using unfamiliar procedures, information and guidance should be obtained from experienced colleagues, as well as from the scientific literature.

### Specific recommendations

*1. Assessment of severity*

1.1 Before assessing the severity of any regulated procedure on the well-being of an animal it is essential that the observer is familiar with the normally accepted behaviour, anatomy, physiology and environmental requirements of the species used, for example growth rate, dietary intake and microbial status.

1.2 Particular attention should be paid to those body systems most likely to be affected by the procedure. With solid tumours this will include ulceration, distension of covering tissues and cachexia. In the case of ascitic tumours, abdominal distension, anaemia and cachexia will be important. Lymphatic involvement from lymphoma and neurological disturbance from intracerebral tumours are examples of special complications arising in specific situations.

1.3 Certain deviations from normal well-being may be difficult to observe, for example induction of anaemia or the development of metastases, and special investigations may be required to detect them.

1.4 Appropriate control animals should always be included, so that the individual effects of the tumour and of any treatment can be distinguished.

*2. Biology of tumours*

2.1 Due consideration should be given to the known biology of the tumour. For spontaneous and transplanted tumours important features will include growth rate, invasion, distension, ulceration, metastases, site, and production of cachectic factors.

2.2 In the case of tumours induced by carcinogens, viruses or genetic manipulation, factors such as method of induction may affect the nature and location of resulting tumours.

2.3 Contamination of tumour cell lines with viruses and other micro-organisms may compromise experimental results, as well as causing an outbreak of disease among laboratory animals. Screening of cell lines for rodent viruses is strongly recommended. For example, Sendai virus is often used to induce cell fusion *in vitro* and is pathogenic to mice and rats. A potential hazard exists for research workers from immune-compromised animals receiving human tumour xenografts which may be contaminated with human pathogens. In such cases, special facilities should be considered for both tissue preparation and animal containment (e.g. flexible film isolators) (see UKCCCR Guidelines for the Xenografting of Human Tumours, 1980).

*3. Humane end points*

3.1 Considerable care should be given to the judicious choice of end point for tumour growth. This should take into account predictable indications of pain, distress or significant deviation from normal behaviour. Unless specified otherwise on the Project Licence, animals should be killed before:
(i) predictable death occurs;
(ii) they get into poor condition;
(iii) the tumour mass becomes over-large, likely to ulcerate or unacceptably limits normal behaviour.

3.2 In the case of local solid tumours, the required information on response to therapy may be obtained by tumour regrowth delay or clonogenic assay, rather than by tumour weight at a given time. Difficulties may arise with this last method because optimum shrinkage of treated tumours may not be achieved before control tumours become excessively large and/or distressing to the host animal. Where such an assay has to be used, the tumour burden should be regulated as indicated in section 3.1.

3.3 The choice of site for solid tumours also requires considerable care, and particular attention should be given to avoidance of sites involving the special senses or where the capacity for tumour growth is limited. Subcutaneous or intradermal growth on the back or in the flank are considered to cause the least distress, while tumours implanted in the footpad, tail, brain and eye will require much greater justification. Distension of musculature is generally painful and this should be considered with intramuscular implants. Extra attention must be paid if multiple sites are used.

3.4 The survival end point should be avoided wherever possible, and its use will require special justification. Where this end point has to be used for animals with ascites or disseminated tumours, particular care should be taken. It is in general unacceptable to wait for predictable death. Animals expected shortly to become moribund should be killed, unless specified otherwise in the Project Licence.

3.5 Difficulties may occur where the effects of anticancer agents on tumour growth are being evaluated. The high toxicity of these agents may combine with the adverse effects of the tumour, but this might be justified by the prospect of a therapeutic remission (as occurs in man). Thus the outcome of such experiments may be uncertain, and uncritical culling would frustrate the purpose of the study. However, where the outcome can be reasonably predicted, animals about to become moribund should be killed.

3.6 No precise quantitative guide can be given as to the acceptable upper limit of tumour burden, since the adverse effects on the host will depend on the biology of the tumour, the site and mode of growth, and the nature of associated treatments. However, tumour burden should not usually exceed 10% of the host animal's normal body weight. It should be emphasized, however, that problems may arise with much smaller tumour burdens.

3.7 With ascitic tumours, including hybridomas, care should be taken to ensure that the volume of ascitic fluid does not become excessive, causing gross abdominal distension, and that solid deposits and cachexia are not allowed to become clinically significant. Ascitic volumes should not usually exceed 20% of normal body weight in mice and rats. Retired breeders are advantageous for monoclonal antibody production, since their abdominal musculature more readily allows larger ascites volumes to be tolerated without discomfort. Ascitic tumours should normally be drained only once. This will minimise the development of solid tumour deposits, bleeding into the peritoneal cavity and cachexia, as well as reducing the risk of introducing an infection. General anaesthesia should always be employed.

3.8 In tumour therapy experiments with adult rodents, it is recommended that weight loss should not normally exceed 20% of the host body weight at the commencement of the experiment. For younger animals, failure to maintain the weight gain seen in untreated control animals should be considered as an indication of toxicity.

3.9 Care should be taken that general housing conditions are appropriate to the known or anticipated condition of the tumour-bearing animal, for example in terms of appropriate bedding, cage structure and accessibility of food and water.

3.10 *Humane end points and other procedures should be refined in the light of experience.*

4. *Examination of animals*

4.1 The frequency with which animals must be inspected for signs of pain or distress and the extent of each examination will be dictated by:

(i) the known biology of the tumour and/or the effects of the inducing agent;
(ii) the effects of any associated techniques;
(iii) the changing clinical status of the animal.

4.2 Rapidly growing or invasive tumours will require more frequent attention, and greater care will be required as the tumour burden increases.

4.3 As a minimum, every tumour-bearing animal should be inspected daily and additional more detailed examinations undertaken as appropriate. The frequency of the latter should be increased during critical periods where the potential for animal suffering may be anticipated. The experimental design should ensure that these do not occur when staff are absent. Particular attention should be given to animals in poor health.

4.4 Appropriate assessment techniques will include: evaluation of overall clinical condition, including appearance, posture, body temperature, behaviour and physiological responses; assessment of food and water intake; weighing to determine changes in body weight (both positive and negative changes compared to controls can be associated with increasing tumour burden); measurements to determine tumour volume or mass; and inspection and palpation to locate the sites of tumour growth, as well as to assess distension, ulceration and compromised mobility.

4.5 Other special examination techniques will be more valuable for specific sites, e.g. breathing rate for lung deposits, neurological disturbance for brain neoplasms, and blood cell counts for leukaemias. Laparotomy or endoscopy may be appropriate in some instances. Estimation of circulating tumour marker substances may also be of value. Autopsy of animals may expose adverse effects undetected by external examinations.

5. *Documentation and publication*

5.1 Researchers are strongly urged, for each tumour model in use in their laboratory, to document the expected behaviour of the tumour and host animal under various experimental conditions, including therapy. They should also document humane end points to limit severity with regard to acute and delayed toxicity and maximal tumour burden, and indicate any particular problems which may be encountered in the use of each model. The appropriate response to such problems should be described and the chain of consultation and responsibility clearly defined. Consideration should be given to the inclusion of a numerical scoring system to facilitate decision-making, e.g. when to contact senior staff or to kill an animal. The guidelines for specific tumour models should be readily available to and agreed between all research and animal husbandry staff involved with that model. Particular care should be taken that all procedures are understood by junior and occasional staff. Researchers are also encouraged to share this information with other groups using the same system, for example when providing a tumour cell line to another laboratory.

5.2 Researchers are encouraged to publish improvements in humane end points in appropriate journals, so as to ensure wide dissemination of the information.

5.3 Encouragement is given to incorporate animal welfare statements into experimental protocols, and in addition to report compliance with these and other appropriate guidelines (including any local ones) when publishing results. Certain journals require this (e.g. the British Journal of Radiology, Cancer Research, and the Journal of the National Cancer Institute).

### Summary and concluding remarks

Researchers have a legal and ethical responsibility to consider the welfare of experimental animals in their care. They must decide whether the use of animals is necessary to answer a particular question, and if so minimise the pain and distress involved. Studies in experimental neoplasia present particular problems. Workers should possess adequate knowledge of the animals and tumour systems to be used. Where unfamiliar procedures are to be employed, information and guidance should be obtained through consultation with experienced colleagues and from the scientific literature. Workers should receive appropriate training and supervision. Pilot experiments should be carried out with small numbers of animals, and numbers should always be restricted to the minimum consistent with the design and purpose of the experiment. Tumour end points should be chosen and refined so as to minimise the adverse effects on the host animal. Survival end points are discouraged. In most instances animals should be killed before they become moribund. Repeated draining of ascitic tumours is discouraged, and a general anaesthetic should be used. All staff should understand their individual responsibilities, and a clear chain of consultation should be established so that prompt action can be taken to deal with any problems that arise. Finally, researchers are encouraged to refine end points in experimental neoplasia and to publish such improvements, to incorporate welfare statements in experimental protocols, and to report compliance with appropriate guidelines in publications.

### References

*Animals (Scientific Procedures) Act 1986.* HM Stationery Office.

*British Council Guidelines on the Use of Living Animals in Scientific Investigations* (1984).

FOWLER, M.E. (1978). *Restraint and Handling of Wild and Domestic Animals.* Iowa State University Press: Ames.

GAY, W.I. (ed) (1965). *Methods of Animal Experimentation*, Volume 1. Academic Press: New York.

*Guidelines on the Care of Laboratory Animals and their Use for Scientific Purposes, Part 1 – Housing and Care.* Royal Society/ Universities Federation for Animal Welfare (UFAW) (1987).

*The Principles of Animal Techniques*, Volume 1. Institute of Animal Technology (1988).

TUFFERY, A.A. (ed) (1987). *Laboratory Animals: An Introduction for New Experimenters.* Wiley: Chichester.

*UKCCCR Guidelines for the Xenografting of Human Tumours* (1980).

**Additional references on animal tumour models and end points**

DENEKAMP, J. (ed) (1980). Quantitation of tumour response: A critical appraisal. *Br. J. Cancer*, **41**, Suppl. IV, 1.

KALLMAN, R.F. (ed) (1987). *Rodent Tumor Models.* Pergamon Press: New York.

KALLMAN, R.F., DENEKAMP, J., HILL, R.P. & KUMMERMEHR, J. (1985). The use of rodent tumours in experimental cancer therapy. *Cancer Res.*, **45**, 6541.

MARTIN, D.S., BALIS, M.E., FISHER, B. & 13 others (1986). Role of murine tumour models in cancer treatment research. *Cancer Res.*, **46**, 2189.

### Appendix

The following examples of tumour systems are given for illustration.

1. *RIF-1 mouse sarcoma.* This is a transplantable sarcoma of C3H/Km mice which is widely used in radiation and chemotherapy studies (Twentyman, *et al.*, *J. Natl Cancer Inst.*, **64**, 595, 1980). It can be maintained in cell culture and is grown *in vivo* as a solid tumour by implantation intradermally in the skin of the flank or intramuscularly in the hind leg. The end points used to determine therapeutic effects on the solid tumour are clonogenic survival, regrowth delay and tumour cure. It is common practice to terminate regrowth delay experiments with leg tumours when the limb diameter reaches approximately 16 mm. At this point the tumour mass is ~3 g or ~10% of the body weight and the host animals are in otherwise normal condition. Growth delay is determined from the time to reach four times the treatment size. Metastases occur late and rarely.

2. *DMBA-induced rat mammary tumour.* (Huggins *et al.*, *J. Exp. Med.*, **109**, 25, 1959). Setting a humane end point with this tumour is more difficult. There is heterogeneity in both the number of tumours which develop and in their relative growth rates, so that individual animals may have widely differing tumour burdens. Close daily monitoring is essential and an overall judgement must be made, based on the aggregate tumour mass, the size and condition of larger tumours and the general health of the animal. While animals may tolerate an aggregate tumour burden of >10% of body weight if there are many small tumours, a single large tumour can lead to rapid deterioration necessitating humane killing of the animal.

3. *MAC 16 mouse colonic adenocarcinoma.* This is a transplantable tumour of NMRI mice which is normally grown subcutaneously in the flank (Bibby, *et al.*, *J. Natl Cancer Inst.*, **78**, 539, 1987). It is of particular interest because it causes progressive cachexia and loss of body weight, beginning at a tumour weight of about 100 mg in a 30 g mouse and increasing over the subsequent 7–14 days. The host mice continue to eat normally over this period. The main difficulty in working with the MAC 16 tumour is the heterogeneity of cachectic response between animals with similar tumour burdens. Because of this, individual animals are weighed at the time of transplantation and then daily thereafter. Mice are killed when the weight loss is between 20 and 40% (maximum). This careful monitoring procedure prevents the occurrence of death due to cachexia.

4. *L1210 mouse leukaemia.* This is normally grown as an ascites tumour and used for the evaluation of anti-cancer agents (Geran *et al.*, *Cancer Chemother. Rep.*, **3**, 1, 1972). The difficulties associated with this model are also shared by other ascites tumour models for which the survival end point has been widely used in the past. Cells (routinely $10^5$–$10^6$) are injected into the peritoneal cavity of C57BL × DBA/2$F_1$ (BD2$F_1$) mice. A direct relationship normally exists between the number of viable L1210 cells injected, or remaining after drug treatment, and the subsequent survival of the animal. Implantation of $10^5$ L1210 cells, with a doubling time of approximately 12 hours in exponential growth, has been shown to produce life-threatening symptoms by the eighth day after implantation. These symptoms are manifested as a marked abdominal distension produced by peritoneal ascitic fluid, dyspnoea, a hunched posture and poor coat quality, particularly a ruffling of the fur, and mild catatonia. As animals approach this phase of tumour growth, twice daily inspection of tumour-bearing animals is necessary to assess morbidity. The therapeutic substance under investigation is normally administered 24 hours after the implantation of the tumour, and may be given at subsequent times. However, the protocol may be modified so as to avoid possible temporal overlap of the toxicity of the substance and the symptoms of morbidity induced by the tumour.

5. *Rodent tumour metastasis models.* Metastasis may be seeded either 'artificially' by intravenous injection of tumour cells, or spontaneously after growth of a solid deposit which can be removed surgically when appropriate. Such models include the B16 and other melanomas and UV-induced fibrosarcomas in mice (Kripke *et al.*, *Cancer Res.*, **38**, 2962, 1978). It may not be necessary to wait until mice develop symptoms of impending morbidity, and the required information may be obtained after humane killing at an earlier stage (see Kripke *et al.*, cited above). Special attention should be directed to detecting signs associated with clinically significant disease in sites particularly susceptible to metastasis, e.g. dyspnoea due to lung deposits.

6. *Chemically-induced colonic tumours in rats.* Tumours of certain internal organs are difficult to detect by external examination. As an example of the use of special diagnostic techniques, colonic tumours in rats can be identified by endoscopic examination (Merz *et al.*, *Hepato-Gastroenterol*, **28**, 53, 1981; Hermanek & Giedl, *Path. Res. Pract.*, **178**, 548, 1984).

7. *Neoplasia in transgenic animals*. Problems may be encountered when oncogenes are inserted or activated, or indeed other genetic alterations are introduced into recipient transgenic animals. In particular it may be difficult to predict the consequences of such genetic changes, which may occur other than in the particular organ of interest. An example of this occurred in transgenic mice carrying a hybrid gene comprising the murine $\alpha$ A-crystallin promoter fused to the coding sequence of the oncogene SV40 T antigen. Not only did the expected lens tumours develop, but in addition several animals developed non-lenticular tumours at various sites throughout the body (Mahon *et al.*, *Science*, **235**, 1622, 1987). Thus special care must be taken to ensure that such associated sequelae are identified and appropriate measures taken.